# Dislocation rates with combinations of anti-protrusio cages and dual mobility cups in revision cases: Are we safe?

**DOI:** 10.1371/journal.pone.0212072

**Published:** 2019-02-07

**Authors:** Tom Schmidt-Braekling, Dorothee Sieber, Georg Gosheger, Jan C. Theil, Burkhard Moellenbeck, Dimosthenis Andreou, Ralf Dieckmann

**Affiliations:** Department of Orthopaedics and Tumor Orthopaedics, University of Muenster, Muenster, Germany; Consorci Parc de Salut MAR de Barcelona, SPAIN

## Abstract

**Background:**

Due to the increasing numbers of revision total hip arthroplasty (THA) procedures being carried out, the frequency of major acetabular defects is also rising. A combination of an anti-protrusio cage and a dual mobility cup has been used in our department since 2007 in order to reduce the dislocation rate associated with complex defects. Although both implants have an important place in endoprosthetics, there are as yet limited data on the dislocation and complication rates with this combination.

**Methods:**

This retrospective study included all patients in whom a Burch–Schneider cage and a dual mobility cup were implanted in our department between 2007 and 2014 and who had a minimum follow-up period of 24 months.

**Results:**

The study included 79 patients with a mean follow-up period of 5.3 years. The implant survival rate was 85% at 65 months. Postoperative dislocation occurred in two cases (2.1%), with the first dislocation taking place within the first 3 weeks in both of these patients.

**Conclusions:**

The present study shows a promising dislocation rate with a combination of an anti-protrusio cage and a dual mobility cup. Particularly in the medium-term follow-up, no further dislocations occurred in the study. A maximum cup inclination of 45° in revision cases was associated with a lower dislocation rate in this group of patients.

## Introduction

Total hip arthroplasty (THA) has been described as representing “the operation of the century” [1]. However, due to increasing life expectancy and more frequent indications for total hip arthroplasty, revision THAs are being required more and more often. Several authors have calculated that a 100% increase in the numbers of THA revisions can be expected by 2026 [2]. THA exchanges are associated not only with increased costs [3], but also with a much higher rate of complications [3] and increased mortality [4]. Due to bone defects, the operations are often more technically demanding, and the reconstruction of the hip cup is often the most challenging aspect involved [5]. On the one hand, the reduced amount of acetabular bone makes it difficult to implant cup systems [5], and on the other the postoperative risk of dislocation of the revision implants is particularly high [6–8]. In the literature, the dislocation rate with primary THA is reported to be 0.2–7.0% [9], while for revisions the rate increases to 10–25% [9]. To address the problem of dislocation, dual mobility cups were introduced as a treatment option by Gilles Bousquet and André Rambert in France in 1974 [10,11]. With complex acetabular defects, however, the surgeon needs to fall back on anti-protrusio cages. In general, polyethylene cups are cemented into the anti-protrusio cages. Several authors have reported that this form of treatment is associated with an increased risk of dislocation [12–16] (Table 1). In order to reduce the risk of dislocation after revision surgery, we have in our own department been implanting a combination of an acetabular support ring and a cemented dual mobility cup since 2007.

**Table 1:** Studies reporting on cemented polyethylene cups in combination with cages.

Study	n	Dislocation rate n (%)	Revision for aseptic loosening n (%)	Revision for septic loosening n (%)	HHS^a^
Regis et al. [15]	18	2 (11.1%)	2 (11.1%)	1 (5.6%)	77
Symeonides et al. [17]	57	1 (1.8%)	6 (10.5%)	0	–
Perka and Ludwig [18]	79/63	4 (5.1%)	1 (1.6%)	2 (3.2%)	74.9
Pieringer et al. [14]	90/67	11 (12.2%)	2 (3%)	1 (1.5%)	73.5
Wachtl et al. [19]	38	2 (5.3%)	0	1 (2.6%)	76
Gill et al. [20]	63	2 (3.2%)	3 (4.8%)	1 (1.6%)	–
Winter et al. [21]	41/38	1 (2.6%)	0	1 (2.6%)	82.6
Udomkiat et al. [16]	64	15 (23.4%)	6 (9.4%)	2 (3.1%)	78.9
Peters et al. [13]	28	2 (7.1%)	0	0	–
Schlegel et al. [22]	298	14 (4.7%)	9 (3%)	9 (3%)	–
Carroll et al. [12]	63	5 (7.9%)	4 (6.3%)	6 (9.5%)	–

^a^ Harris hip score.

Despite the increasing numbers of patients who require implantation of a cage due to the acetabular defect situation, the data are still as yet limited for the dislocation rate after implantation of a combination of a Burch–Schneider cage and a dual mobility cup. The aim of the present study was to review the postoperative clinical results and complications—particularly the dislocation rate—associated with this combination of implants.

## Materials and methods

### Study group

The study was proved by the local ethics committee of the University of Muenster, Germany (Approval N° 2017-428-f-s). Patient consent was not deemed necessary for retrospective analysis since we used routine patient data.

This retrospective study included all patients in whom a Burch–Schneider cage and a dual mobility cup were implanted in our department between 2007 and 2014 and who had a minimum follow-up period of 24 months. Implantation of the Burch–Schneider cage was indicated in cases of extensive cup base defects and an unstable caudal cup edge. All patients who had to receive a tumor prosthesis or push-through prosthesis due to a defect situation in the shaft area, or who were undergoing surgery due to a malignant disease, were excluded.

The operation was carried out in 97 consecutive patients over the stated period. Five patients died before the end of the 24-month follow-up period; in six patients, the prosthesis was explanted again before the end of the 24 months due to aseptic (n = 3) or septic events; and seven patients did not attend for the 24-month follow-up visit and could no longer be contacted during the subsequent course, even by phone. A total of 79 patients with a minimum follow-up period of at least 24 months thus remained in the study, representing a follow-up rate of 92.8%.

All of the operations involved revision endoprosthetic procedures, including both cup revisions alone and also complete THA exchanges. Data on demographic characteristics, body mass index (BMI), side of surgery, diagnosis, type of surgery, preoperative C-reactive protein (CRP) values, postoperative Harris hip scores, und radiographs were collected.

### Patients

The study group consisted of 55 women and 24 men. Forty-six right hips and 33 left hips were operated on. The patients’ mean age at the time of surgery was 68.5 years (range 41–87 years), and their mean BMI was 26.8 kg/m^2^ (range 18.6–41.5 kg/m^2^). Sixty-four of the operations involved aseptic revisions; 10 were reimplantations in the setting of a two-stage exchange with periprosthetic infection; and in five patients the reason for surgery was recurrent dislocation of the existing prosthesis. The mean number of previous operations in the overall group was 2.3 (range of prior operations 1–6). A complete THA exchange was carried out in 33 patients, while in the other patients only the existing hip cup was exchanged.

The dislocation and complication rates were calculated on the basis of all patients in the group (n = 97), regardless of whether the 24-month follow-up period was completed.

### Implants

Since 2007, all patients with higher-grade acetabular defect situations have been treated with a combination of a Burch–Schneider cage (Burch–Schneider Cage; Zimmer, Warsaw, Indiana, USA) and a cemented dual mobility cup. Up to the end of 2012, the cemented dual mobility cup that was implanted (in 60 hips) was the Avantage dual mobility cup (Biomet, Warsaw, Indiana, USA). Starting in 2013, we used the EcoFit 2M cup (EcoFit; Implantcast, Buxtehude, Germany), in 19 hips. Cup base regeneration treatment was carried out in 63% of the patients. Both cancellous bone and bone replacement material were used for this.

### Clinical and radiological evaluation

The patients received clinical and radiographic check-up examinations at the routine postoperative visits after 3 and 9 months and then at annual intervals during the subsequent course. The clinical Harris hip score [23] was also calculated at the follow-up visit after 24 months.

The American Academy of Orthopaedic Surgeons (AAOS) classification [24] was used to assess the acetabular defect situation on the basis of the preoperative radiographs and the surgical report (Table 2).

**Table 2:** American Academy of Orthopaedic Surgeons (AAOS) classification of the acetabular defect situations in the 79 patients.

AAOS	Patients n (%)
Type I	4 (5%)
Type II	4 (5%)
Type III	57 (72%)
Type IV Type V	14 (18%) 0

The postoperative component positioning was assessed using a picture archiving and communication system (PACS). The cup inclination (CI) was measured in relation to the horizontal inter-teardrop reference line on pelvigraphy views with anteroposterior beam projections. Since the dual mobility cups used in our study are not hemispheric, we measured the cup inclination with the lateral rim as the most cranial measurement point. The target zone for inclination was defined as 30–50°, in accordance with the Lewinnek safe zone [25]. Definitive loosening was defined as follows, based on the descriptions presented by Gill et al. [20]: migration of the acetabular support ring by more than 5 mm, fracture of at least one screw, or fracture of the acetabular support ring or part of it.

### Statistical analysis

Subject privacy and confidentiality was maintained through the storage of study data in a password-protected computer database maintained by the first author. Each subject was assigned a unique study number for identification in the study database. Data collection and statistical analysis were performed using Excel (Microsoft Corporation, Redmont, Washington, USA) and SPSS version 25 (IBM Corporation, Armonk, New York, USA). Descriptive statistics were used to analyze the distribution of data, and means and ranges were calculated for normally distributed data; medians and interquartile ranges are given for data without a normal distribution. Survival was evaluated using Kaplan–Meier curves, and differences and influencing factors were assessed using the chi-squared test and log rank test. Statistical significance was defined as *P* ≤ 0.05.

The implantation date was defined as 0, and the end points considered were implant failure resulting in loss of implant, and death. Implant survival and last follow-up were calculated.

## Results

**Fig 1:**
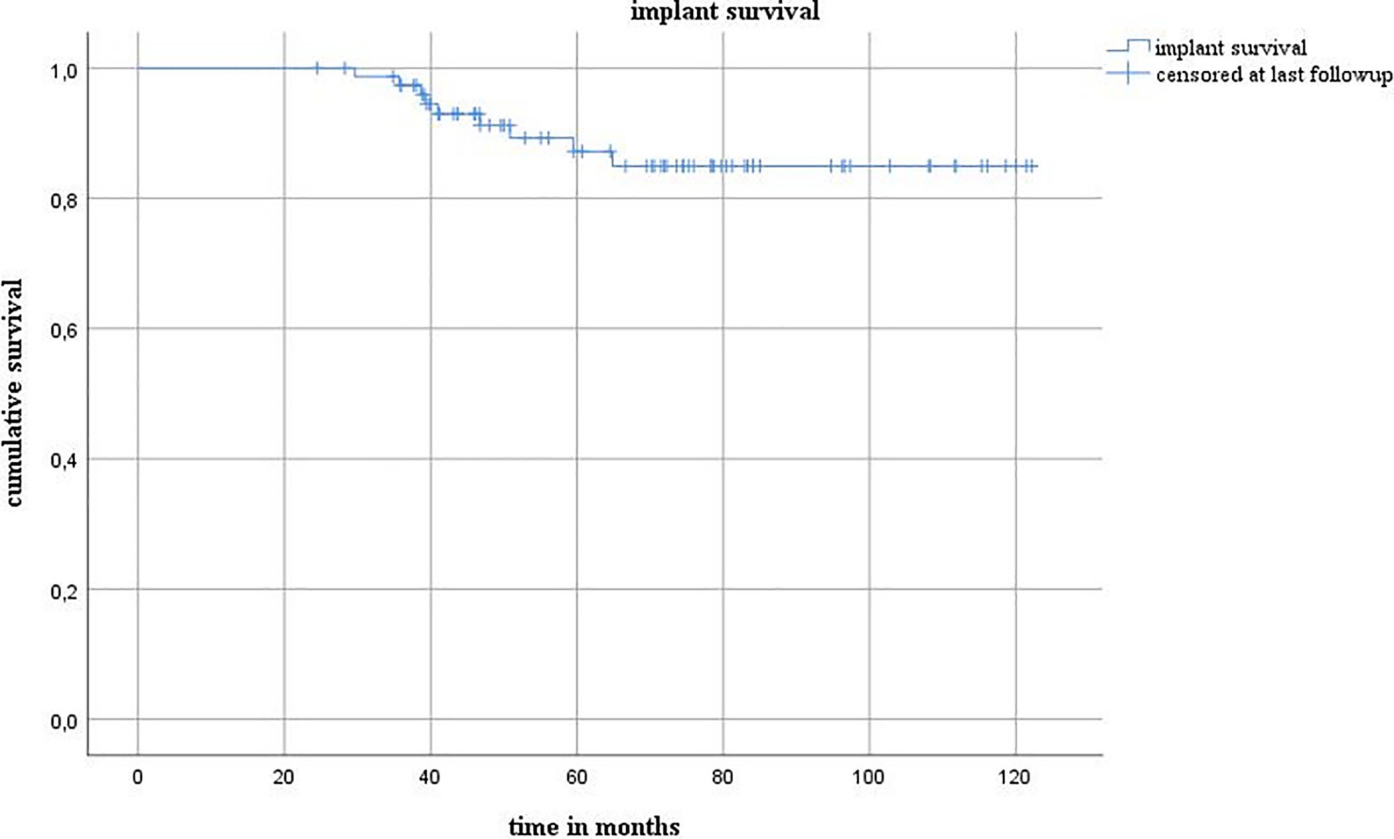
Implant survival.

The mean follow-up period was 5.3 years (range 2.0–10.2 years). The implant survival rate was 85% at 65 months (Fig 1). Postoperative dislocation occurred in two cases (2.1%), and the first dislocation occurred within the first 3 weeks in both of these patients (Table 3).

**Table 3:** Further details of the two patients in whom dislocations occurred.

	Patient no. 1 (female)	Patient no. 2 (female)
Age on day of surgery (y)	75	84
Previous operations (n)	3	2
AAOS^a^ score	3	1
Indication for revision surgery	Aseptic loosening with subsequent recurrent dislocations	Aseptic loosening
Implant	BSC^b^/Avantage cup	BSC^b^/Avantage cup
Size of liner/head	Cup size 46, head size 22.2	Cup size 52, head size 28
Cup inclination	53°	56°
Dislocations	2, then shaft revision, then 1	1
Time of dislocation	4th postop. day, 4.3 months postop., 2 months postop.	24th postop. day
Procedure	2 × closed repositionings, then shaft exchange, 1 × closed repositioning	1 × closed repositioning
Harris hip score	60	57
Follow-up (months)	41.6 (then died)	83.3

^a^ American Academy of Orthopaedic Surgeons

^b^Burch–Schneider cage.

**Fig 2:**
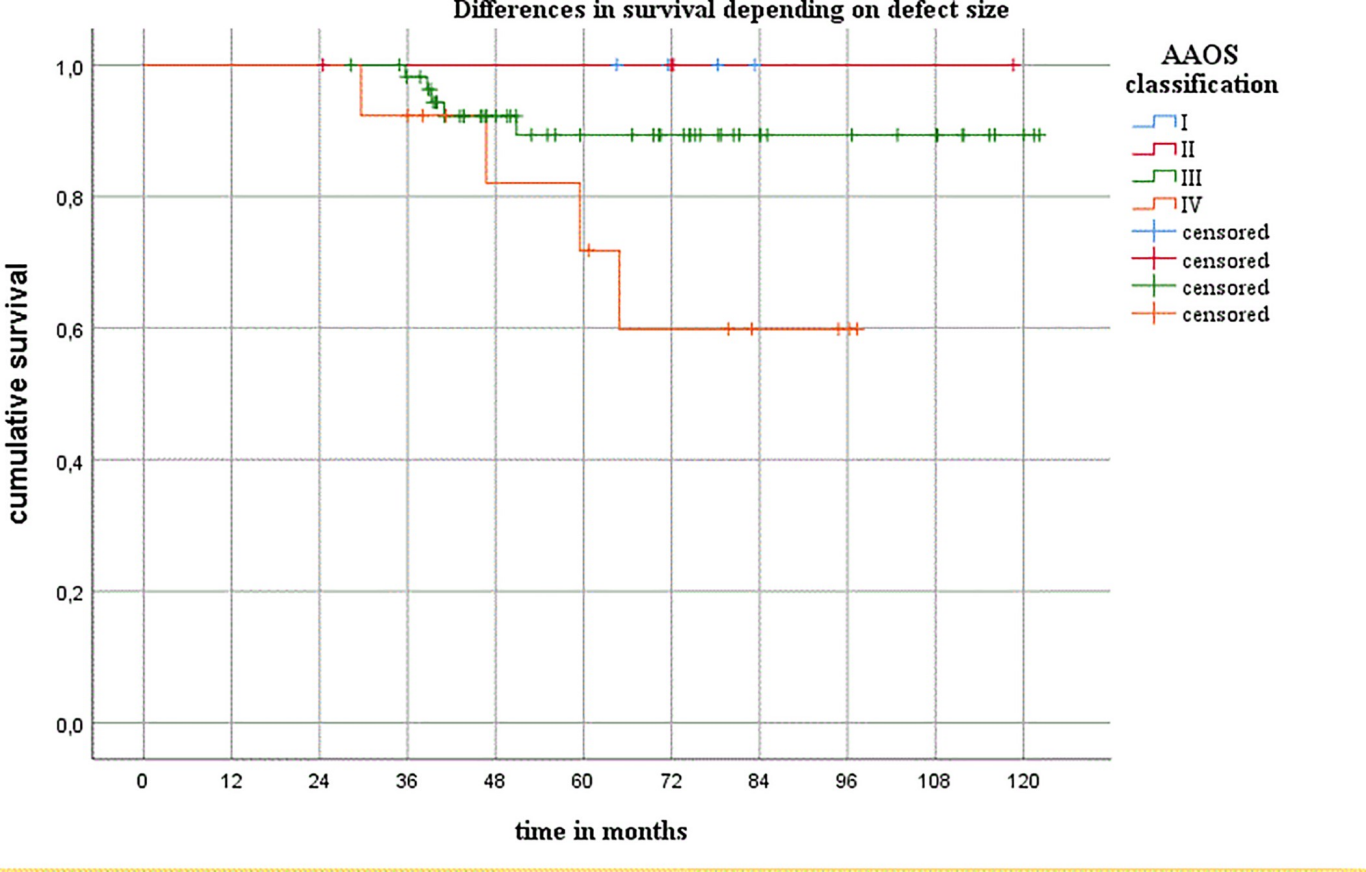
Differences in survival relative to the defect size. AAOS, American Academy of Orthopaedic Surgeons.

**Fig 3:**
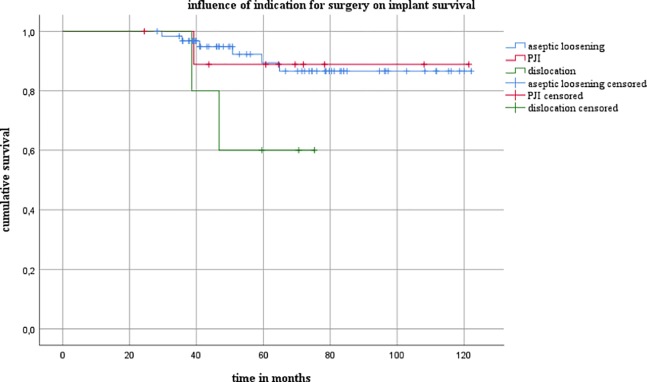
Influence of the indication for surgery on implant survival.

Eight patients died during the follow-up period. No repeat revision procedures or hip dislocations occurred up to the time of death in any of these patients. Nine patients had to undergo a repeat cup revision during the follow-up period. Periprosthetic infection was noted in five of these patients, and aseptic loosening was the reason for the revision in four of them. The mean number of acetabular defects as defined in the AAOS classification was 3.0 (range 1–4). The defect size was found to have a significant influence on implant survival when AAOS III and IV defects were compared (*P* = 0.047) (Fig 2). It was also examined whether the indication for surgery (aseptic or septic exchange, or recurrent dislocation) influenced implant survival. There were no statistically significant differences in implant survival between patients who were treated for aseptic loosening and patients in whom Burch–Schneider cages were used during reimplantation surgery in two-stage revision procedures for periprosthetic joint infection (PJI) (Fig 3). There was a trend toward shorter implant survival periods in patients who were treated for dislocation in comparison with those with aseptic loosening (*P* = 0.065). There were no significant differences between patients who were treated for PJI and those who were treated for dislocation.

### Dislocation rate

In the first patient, surgery was carried out due to aseptic loosening with subsequent recurrent dislocations. Following the index operation, the first dislocation occurred due to trauma on the fourth postoperative day. This was followed by another dislocation, which was also reduced using a closed procedure. During the subsequent course, the shaft was then exchanged. A further dislocation occurred postoperatively, which was again reduced with a closed procedure. No further dislocation events occurred up to the time of the patient’s death (follow-up period 3.5 years). The cup inclination was 53°.

The second patient underwent a cup exchange to a Burch–Schneider cage with an Avantage cup due to aseptic loosening. A dislocation occurred 24 days after the operation. Following closed repositioning, no further dislocations occurred during the subsequent course (current follow-up period 6.9 years). The cup inclination was 56°.

In the overall group, five patients underwent surgery due to preoperative dislocation events. The hip prosthesis no longer dislocated in any of these cases after implantation of the combination of an acetabular support ring and a dual mobility cup.

### Complications

The most frequent intraoperative complication was femoral fracture, which occurred in six cases (6.1%). Intraoperative bleeding occurred in three prosthesis implantation procedures (3.1%); one ischial and one acetabular fracture occurred intraoperatively in one patient each (1.0%); and the femoral nerve was damaged during surgery in one patient.

Postoperative thromboses developed in two patients (2%); paresis of the peroneal nerve was observed in one case (1%); and adipose tissue necrosis with wound healing disturbances occurred in one patient. Five patients had to undergo revision procedures due to periprosthetic infection during the later course. The revisions were carried out at a mean of 4.1 years postoperatively (range 2.5–5.4 years). Revision procedures were carried out due to aseptic loosening in four patients, with the mean time to revision amounting to 3.4 years (range 3.0–3.9 years). Overall, the prostheses required revision in nine patients—a revision rate of 11.4%.

### Cup inclination

The mean inclination angle was 40° (range 22–58°). Eighty-five cups (87%) were implanted with angles of between 30° and 50° and were therefore within the Lewinnek safe zone [25]. An inclination angle of < 30° (range 22–28°) was present in six hips, and in six cases the angle was greater than 50° (range 53–58°).

The two dislocated hips had inclination angles of 53° and 56°, so that both of these cups had angles beyond the upper limit of the Lewinnek safe zone.

### Harris hip score

A postoperative Harris hip score (HSS) was calculated after 24 months. If there was more than one HSS available for a patient, the latest one was always used for the present data analysis. The mean postoperative HSS score was 73.0 points (range 24–99).

## Discussion

The combination of an acetabular support ring and a dual mobility cup is a highly promising option in relation to dislocation and complication rates in patients with acetabular bone defects.

Despite the substantial number of large acetabular defects in the overall patient group (90% with AAOS grades III and IV), the dislocation risk in the group, at 2.1%, was similar to or better than that in comparable studies in which a dual mobility cup was not used during the implantation of acetabular support rings [14–16].

For comparison, there have been numerous studies reporting on a combination of a cage with a cemented polyethylene cup. The highest dislocation rate (23.4%) was reported by Udomkiat et al. [16]. This high rate was explained by the authors as resulting from a soft-tissue imbalance and weakness in the abductors. Pieringer et al. also reported a dislocation rate of 12.2% with a combination of a Burch–Schneider cage and cemented polyethylene cups [14]. By contrast, however, a few research groups have also reported lower dislocation rates. Symeonides et al. report only one dislocation (1.8%) in a group of 57 patients who received a combination of a Burch–Schneider cage and a cemented polyethylene cup [17]. However, the study also included primary THAs (14% of cases). In comparison with revision procedures, primary hip THAs are associated with a lower dislocation rate [6–8].

Revisions with cages and cemented dual mobility cups may also be associated with higher rates of dislocation, however. Schneider et al. analyzed a series of 96 revisions in which cages and dual-mobility cups were used [26]. The authors reported a dislocation rate of 10.4% with a mean follow-up period of 41 months (range 1–101 months). Despite the relatively large number of postoperative dislocations, the authors recommend a combination of cage and dual mobility cup as a way of containing the dislocation rate. By contrast, Civinini et al. reported a dislocation rate of 0% in a group of 33 patients with isolated acetabular revisions, with a minimum follow-up period of 2 years [27]. The study included 24 patients in whom a dual mobility cup was cemented into an anti-protrusio cage.

However, other factors are also known to increase the risk of dislocation in revision cases. On the one hand, Wetters et al. showed in a retrospective study that large acetabular defects (Paprosky IIIA/IIIB) represent a clear risk for postoperative dislocation (OR 1.522) [28]. The authors considered that abductor weakness and the difficulty of restoring the former center of rotation were responsible for the increased dislocation rate. Dislocations occurred in 9.8% of cases in the study (in 1152 revision THAs).

On the other hand, several research groups have shown that patients who undergo surgery for recurrent dislocations have a clearly increased risk for developing a persistent tendency to dislocate [28–31]. Jo et al. reported a recurrent dislocation rate as high as 35% in patients who had undergone revision for recurrent dislocations [30]. Postoperative dislocations are often associated with poor soft-tissue conditions and poor bone stock [28,29]. A recent study by Chalmers et al. reported promising results, over a short-term follow-up period, with the implantation of dual mobility cups in revision procedures carried out due to recurrent dislocation [32].

A third risk factor for postoperative dislocation is present in patients who undergo two-stage exchanges in which a periprosthetic hip infection develops. In a study including 169 patients who underwent two-stage revisions, Sanchez-Sotelo et al. reported a dislocation rate of 11% (n = 19) after reimplantation of the hip endoprosthesis [33]. Of these 11 patients, 42% (n = 8) had to undergo revision procedures due to recurrent dislocations.

### Limitations

This study has the following limitations. (1) The first limitation to be mentioned is the study’s retrospective nature. (2) In addition, all patients in whom a Burch–Schneider cage has been implanted since 2006 have also been treated using a dual mobility cup. Since the dislocation rate results were highly promising from the very start, no more polyethylene cups have been cemented into the Burch–Schneider cages since that time. A control group is therefore lacking. (3) The third limitation to be mentioned is the inclusion of all patients, regardless of their age or comorbidities. Furthermore (4) we related the dislocation rate to all patients in our study, regardless of the follow up. This could be a possible bias of this study, since the long term dislocation rate might be higher.

## Conclusions

The present study shows that a promising dislocation rate can be achieved with a combination of an anti-protrusio cage and a dual mobility cup. Particularly in the medium-term follow-up, no further dislocations occurred in the group of patients included in the study. A maximum cup inclination of 45° in revision cases was associated with a lower dislocation rate in this group of patients.
